# Teaching in radiation oncology: now and 2025—results of a focus group with medical students

**DOI:** 10.1007/s00066-022-01997-0

**Published:** 2022-09-05

**Authors:** Philipp Linde, Marie Klein, Frauke Lang, Simone Wegen, Cordula Petersen, Hendrik Dapper, Jiaqi Fan, Eren Celik, Simone Marnitz, Christian Baues

**Affiliations:** 1 Department of Radiation Oncology, Cyberknife and Radiation Therapy, Faculty of Medicine and University Hospital of Cologne, University of Cologne, Kerpener Str. 62, 50937 Cologne, Germany; 2 Department of Radiotherapy and Radiation Oncology, University Medical Center Hamburg-Eppendorf, Martinistr. 52, 20246 Hamburg, Germany; 3 Department of Radiotherapy and Radiation Oncology, Public Hospital of Bielefeld, University Medical Center East Westphalia-Lippe, Teutoburger Str. 50, 33604 Bielefeld, Germany

**Keywords:** Medical education, Radiation oncology teaching, Curriculum, Radiation oncology, Medical studies

## Abstract

**Purpose:**

In Germany, the new Licensing Regulations for Physicians 2025 (Ärztliche Approbationsordnung, ÄApprO) define a binding legal framework on the basis of which medical faculties modernize their curricula. Since 2015, the National Competence Based Learning Objectives Catalogue for Medicine 2.0 (Nationaler Kompetenzbasierter Lernzielkatalog 2.0., NKLM) formulates competencies and learning objectives to be achieved in the course of studies as curriculum orientation for the medical faculties. In addition, about 80% of the areas of a new core curriculum are to be made compulsory. A needs analysis in the target group of students has not yet taken place for the subject of radiation therapy (RT*) *or radiation oncology (RO). This study therefore surveys the experiences and requirements of students regarding medical education in RT.

**Methods:**

Qualitative single-center study using a semistructured in-depth focus group with 11 medical students (20–26 years; 6 female, 5 male) was conducted. Brainstorming sessions were conducted in small groups and individually; oral contributions were recorded, transcribed, and analyzed using qualitative content analysis according to Mayring. Results were compared with the content of the future curriculum and reviewed for congruence with current expert recommendations of the German Society of Radiation Oncology (Deutsche Gesellschaft für Radioonkologie, DEGRO).

**Results:**

The plans to develop a longitudinal and practice-oriented curriculum was positively received by students. Specifically, students wanted to introduce the basics of RT as an early link to practice in preclinical teaching units. The necessary acquisition of communicative skills should also be taught by lecturers in RO. Methodologically, regular digital survey tools for self-monitoring, discussion rooms, and problem-based learning were named. In the perception of students, the subject appears underrepresented in relation to its relevance in the multimodal therapy of oncological diseases.

**Conclusion:**

Results of the needs analysis for the subject of RT are consistent with ÄApprO, NKLM, and DEGRO. Moreover, they complement them and should be considered in the curriculum development of Masterplan Medical Education 2020 (Masterplan Medizinstudium 2020). The results contribute to high-quality and target-group-oriented medical training in the subject of RT, increased visibility, and thus early bonding of future physicians to RO in Germany.

## Introduction

The modern physician image extends by far beyond mere knowledge and skill. A variety of personal and practical requirements complement the role and should therefore already be considered in the training of future physicians [1, 2].

One in two cancer patients will receive radiation therapy (RT) during the course of disease [3]. As a result, radiation oncology (RO) plays a major role in the steady improvement of care for cancer patients of all ages with a wide variety of tumors [4]. However, this relevance does not seem to be reflected in the current curricula of medical studies at faculties in Germany [5]. Furthermore, RO plays a subordinate role in the awareness of students and many prejudices and uncertainties persist regarding this specialty [6, 7]. This indicates the importance of a nationwide curriculum with operationalized learning objectives, developed according to high quality standards.

The universities determine the details of cross-sectional teaching in their study regulations [8]. The teaching should be topic-related, interdisciplinary, and oriented towards the subject matter. RO is part of cross-sectional subject 11 “imaging procedures, radiation therapy, radiation protection” (Querschnittsbereich 11: Bildgebende Verfahren, Strahlenbehandlung, Strahlenschutz, QB 11), which also includes radiology and nuclear medicine [5].

In 2017, the Health and Science Ministers of the federal and state governments passed a resolution to revise and modernize medical studies in Germany. The Masterplan Medical Education 2020 (Masterplan Medizinstudium 2020) focusses on practical relevance and the acquisition of social and communicative competencies in the formation of future physicians [9]. The explicit contents of the core curriculum are specified in the National Competence Based Learning Objectives Catalogue for Medicine 2.0 (Nationaler Kompetenzbasierter Lernzielkatalog Medizin 2.0, NKLM), which has been revised since 2015 in an ongoing process in cooperation between Medical Faculty Association of the Federal Republic of Germany (Medizinischer Fakultätentag der Bundesrepublik Deutschland, MFT) and the Society for Medical Education (Gesellschaft für Medizinische Ausbildung, GMA) [10]. The NKLM and Subject Catalog (Gegenstandskatalog, GK) of the Institute for Medical and Pharmaceutical Examination Questions (Institut für medizinische und pharmazeutische Prüfungsfragen, IMPP) complement each other (“constructive alignment”) [10]. The GK specifies contents for the written part of the second section of the medical examination in Germany. The updated edition 5.1, which will become relevant in the coming months, also defines medical roles and activities of graduates [11].

The restructuring of the curriculum necessitated a new version of the Licensing Regulations for Physicians (Ärztliche Approbationsordnung, ÄApprO), which is to be implemented at all German universities by 2025 [12]. The Masterplan Medical Education 2020 allows students to individually design areas of their studies according to their individual interests (up to 20%) [10]. Thereby, the implementation in detail is left to the faculties themselves. This provides an opportunity to involve all parties of the faculty in the renewal of teaching. In this regard, the German Society for Radiation Oncology (Deutsche Gesellschaft für Radioonkologie, DEGRO) has announced recommendations on the content and scope of how RO should be represented in the new curriculum [5].

A medical studies curriculum is a pedagogical design that summarizes the educational focus of a subject with operationalized learning objectives. Tangible learning goals and competencies are formulated, and observable characteristics of future physicians are named. Kern et al. distinguish six phases in curriculum development [13]. In addition to the needs analysis of experts (step 2), the needs analysis of learners as a target group is an indispensable step while developing a curriculum [14]. Therefore, we see the necessity in both: to optimize teaching in RO and to integrate students’ needs in further development of medical curricula.

To the authors’ knowledge, this is the first prospective study to survey students’ experiences and requirements of medical education in RO in Germany. It analyzes whether the actual recommendations reflect the assessment of the students and—in addition—which modules could be meaningful and useful from the students’ perspectives.

## Materials and methods

### Study design and ethics

A qualitative study design was used with semistructured, face-to-face in-depth focus group interviews with 11 medical students at the University of Cologne. This study followed the consolidated criteria for reporting qualitative research [15]. Ethical approval was obtained from the Ethics Commission of the Faculty of Medicine of the University of Cologne (Reference Number 21-1042).

Participation was voluntary and opened to students of all years. An invitation to participate was sent to members of the medical faculty student council as well as students who had previously completed an internship in RO or participated in voluntary courses. At the outset, participants were asked to introduce themselves by stating their name, semester, and motivational reason for participating in the focus group. The study team then provided a brief overview of the work of a RO specialist, as well as the courses and lectures taught in QB 11. This was followed by flipchart-based mind mapping of praise and criticism of teaching in RO at the Medical Faculty of the University of Cologne in small groups of 3 to 4 students each. In addition, the participants collected competencies that—in their opinion—a physician needs in RO and whose acquisition should therefore be part of the teaching concept in this subject.

In another following group work, brainstorming sessions were conducted to resolve previously collected comments. The results were presented and discussed in the whole group. This section was tape-recorded and later transcribed verbatim. Finally, participants took notes of method-related aspects that they remembered as particularly positive during their studies. This was followed by a second run in which the question was repeated with special attention to the digital formats introduced during the coronavirus disease 2019 (COVID-19) pandemic. If necessary, the research team guided the medical students back into the discussion to focus on RO if they seemed to be off track in terms of content/topic. (Side-)notes were taken during and at the end of the interviews by the research team. The group distribution specified by the study team attempted to include and unify as many student perspectives as possible in the results. In the evaluation, no prioritization of the results was made. The suggestions were based exclusively on the opinions and wishes of students. They were not checked for feasibility in the university context.

The results were analyzed with a qualitative content analysis. The subject of the analysis was the material collected during the interactions within the focus group. The objects and transcripts were analyzed according to explicit rules under a theoretically defined question. To draw conclusions from the transcript of the focus group, statements were deductive–inductively assigned to categories for content-related or method-related aspects [16–18]. Finally, the results were compared with the contents of NKLM, GK, and latest recommendations of the DEGRO experts [5].

## Results

### Participants

Eleven students from the University of Cologne participated in the focus group (20–26 years; 6 female, 5 male) in December 2021. Many were members of the Medical Student Council and already had experience with curriculum development in working groups. Most participants attended clinical semesters and had already completed the courses in RO. Their motivation to participate in the focus group was to improve medical teaching in general. Two participants attended preclinical courses and had no previous contact with teaching in this subject. Thus, they had a personal interest in contributing positively to teaching for future semesters. The focus group session lasted 180 min (not including follow-up on the collected data). It was also initially noticeable that some participants did not differentiate precisely between the individual subjects (radiology, nuclear medicine, RT) taught in QB 11. They described their experiences with QB 11 in general or recalled specific aspects of other subjects, e.g., teaching in radiology.

### Method-related aspects

In the content analysis of the students’ brainstorming for methodological aspects, the desire for practical methods such as case studies with simulated patients or interactive case discussions in medical education emerged across all semesters. Medical students mentioned observing the daily routine on the wards, during medical talks and their simulation, as an optimal preparation for their later work in the hospital. From the students’ perspective, regular digital self-checks, for example at the end of a lecture, or problem-based learning (PBL) in small groups, have proven to be effective in preparing for exams in a most time-efficient and targeted manner. In addition to the encounter with real patients, example cases highlighted by characteristic attributes were particularly memorable. Furthermore, fictitious patient names such as Wilhelm Conrad Röntgen, for example, were named by students as memorable even beyond further semesters of study.

Especially due to the COVID-19 pandemic, everyday life at universities has been increasingly and necessarily characterized by digital teaching formats. Participants’ opinions on this were heterogeneous. Generally, voting sessions in lectures with many students during the lecture or exemplary questions on previously discussed contents at the end were stated as highly effective to lead to more interaction between lecturer and students. Hybrid lectures, which can be attended both face-to-face and via self-study using video and audio recording, were named as useful to allow the students to manage their time individually and thus have more leeway to take their own needs into account. In addition, students would like to see the integration of available video materials and publicly accessible online courses for self-studying to minimize class time with purely face-to-face instruction. The principle of ZOOM’s (San José, CA, USA) breakout sessions received mostly positive feedback. Here, partial aspects of a topic can be discussed with a few participants of a larger group either in attendance or via digital communication portals [19]. Table 1 provides a detailed overview.

**Table 1:** Method-related recommendations of the focus group with students

*Practical relevance*
Joint development of contents on the basis of case studies
Exercises with acting patients
Patient interviews conducted by students
Interactive seminars for case discussion of smaller topics
Case studies with memorable patients, e.g., through striking names (Wilhelm Conrad Röntgen) or salient characteristics
Practical application of theoretical knowledge, e.g., in the context of anamnesis, clarification discussions, physical/diagnostical examination techniques
*Motivation by interaction and variety*
Problem-based learning (PBL)
Break-out sessions
Inverted classroom
Self-checks at the end of lectures
Sample questions/exams to highlight take-home messages and to prepare for exams
Combination of different formats: recorded lectures or videos; short videos for preparation and interactive zoom seminars for discussion
*Use of digital tools*
Hybrid events
Lecture recordings with Opencast
Voting during lectures via digital polling tools
Usage of eLearning videos, publicly accessible digital formats, or topic-related current teaching videos
Break-out sessions: *discussions on individual aspects of a topic with a few participants of a larger group *[19], Hybrid events: *participation possible in presence; audio and image recording for self-study *[48], inverted classroom: *acquisition of theoretical knowledge in self-study for preparation, practical application in seminar form for reduction of teaching units in frontal teaching *[38]*,* PBL *problem-based learning*: *self-directed learning by focusing on problems to promote understanding *[49]*, *Opencast: *open source software for planning, recording, and publishing audiovisual *[50]

### Content-related aspects

#### Required competencies in RO from the students’ perspective

When asked about their perceptions, the focus group participants considered social skills and empathy to play a greater role for a physician in RO. These are necessary, for example, for conducting educational discussions and empathetic communication of the therapy plan during or within the first consultation. In addition, learning to work in an interdisciplinary manner was seen as essential for therapy planning and implementation. Basics in medical physics as well as the reporting of radiological examinations were assumed, just as RO expertise. The combination of different competences is necessary for the multimodal care of oncological patients. The students wish these competences to be taught early during the medical studies including theoretical frameworks and to also be applied frequently.

#### Contents and structure of a student-created curriculum

Students supported the idea of a longitudinal orientation of the curriculum. In the opinion of the interviewed students, basics of RO should already be integrated into subjects such as medical physics and biology. Furthermore, overlap with immunology content, such as repair mechanisms for cellular damage caused by radiation, should be considered. The structure of QB 11, which in addition to RT also includes radiology and nuclear medicine, was a subject of discussion. Some felt the combination was counterproductive because without a common guideline, the subjects would coexist rather than form a collaborative cross-section. Others saw this as an opportunity: once individual specialties were clearly defined, joint basic lectures could minimize redundancy. Seminars with coordinated content and held jointly, for example in the form of a simulated tumor conference, would allow training in interdisciplinary work and presentation of patient data to colleagues, in addition to teaching of content.

Interactive lectures with case studies should cover contents of the entire QB 11 and, thus, contribute to a practical and varied teaching. Regarding the support of oncological patients, it is not only important to recognize side effects of RT early during therapy and to treat them adequately, but also to gain empathic insight into psychological aspects. A visiting day to an oncology ward could link practical courses in different subjects. By using patient files, students could work out relevant information across subjects and present it to their fellow students in small groups. Finally, students named and supported a need for basic information, for example, on radiation protection for medical staff. Table 2 provides a detailed overview and comparison between results of focus group and DEGRO recommendations.

**Table 2:** Content-related results of the focus group with medical students. One-on-one comparison of student recommendations with DEGRO recommendations [5]

*Student recommendations for teaching content in RT*	Format proposal	Recommended by DEGRO^a^
**Integration of the basics of RO in preclinical subjects**		
Effect of radiation on human cells	Biology lecture	Yes
Radiation sensitivity of different tissues	Biology lecture	Yes
Repair mechanisms of radiation damage	Biology lecture Immunology lecture	Yes
Basics of radiation	Physics lecture	Yes
**Joint lectures of QB 11**		
Origin, conduction, use of radiation	Joint basic lecture	Yes
Function of X‑ray equipment	Joint basic lecture	Yes
Clear definition of different areas of responsibility of the disciplines in QB 11	Joint introductory session, case examples, visit to clinical wards, demonstration of tumor board (e.g., video recording)	Yes
Training interdisciplinary working	Preparation of a therapy plan, simulation of a tumor board with delegated roles	Yes
*Case studies from the entire QB 11 spectrum:* *imaging and its findings, treatment planning,* *possible side effects with treatment, communicating with patients*	*Discussing the course of a case in a realistic order in seminars with 10–15 participants*	
**Interdisciplinary oncology courses**		
Patient examples/acting patients for the most common tumor entities in RT	RT digression during lectures in other disciplines	Yes
Multimodal care of oncological patients	Holistic view of case studies	Yes
Basic palliative patient care	Integration in palliative medicine lecture	Yes
**Communicative and social skills**		
Patient interviews, training for Breaking Bad News, educational talks, communicating a therapy concept in understandable language	Integration in communication training, simulation of explanatory talks, group exercises, role plays, case studies	Yes
*Empathizing with the patients’ point of view*	*Observation at medical consultations early in the study: debriefing and reflection*	
**Key knowledge**		
What should everyone know across all disciplines? What is the typical course of RT? What are the effects on patients?	Knowledge of the most common and relevant side effects: symptoms, frequency, duration, treatment options	Yes
Know the most common and relevant side effects: symptoms, frequency, duration, treatment options	Simulation of a medical conversation in partner work or with acting patients	Yes
**Advanced courses**		
Deepening of relevant contents	Interactive seminars and clinical internships	Yes
Handling complex case studies	Group work on different cases with materials provided or possibility to research, presentation in the group, simulation of a tumor board in seminar groups, interactive handling of a case example	Yes
Working with patient cases	Internships on hospital wards, joint internships with oncology or palliative medicine, elaborating patient files	Yes
*Delineation of the areas of responsibility of radiation oncologists and medical physicists*		
*RT outcome of single cancer entities*		
**Radiation protection**		
Information on radiation damage among medical staff and measures for the prophylaxis of radiation damage	Demonstration of correctly worn radiation protective clothing, visiting medical equipment	Yes

*RO* radiation oncology, *RT* radiation therapy, *QB 11* cross-sectional subject 11 “imaging procedures, radiation therapy, radiation protection”

^a^Recommendations named by DEGRO

## Discussion

This prospective single-institution analysis shows that the students have substantial, and mostly also methodologically realizable ideas of teaching–learning methods in RO. From the medical students’ point of view, RO is currently underrepresented in the curriculum. Students would like to see a clear concept for teaching QB 11 without redundancies. Especially in the acquisition of communicative competencies, RO should be integrated more profoundly in the future. Finally, the results of the needs assessment in the group of medical students in the subject of RO agree with the expert suggestions of ÄApprO, NKLM, and DEGRO.

The experiences of the target group provide valuable information, resulting in an optimal concept for the implementation of the ÄApprO for teaching in RO. Ranasinghe et al. have already shown how qualitative feedback from students highlights certain key areas that require special attention when creating the curriculum [20]. It also provides space for possible creative solutions as proposed and perceived by students. Up to now, teaching of RO in Germany has been heterogeneous in terms of methods and content [21]. According to ÄApprO, an intensification of the longitudinal structure is considered necessary by the focus group participants and DEGRO experts alike [5]. Despite the clear separation in QB 11, teaching must be coordinated. A common guideline and agreements among lecturers minimize redundancies and thus improve the learning experience for both lecturers and students. It is necessary to define exactly what knowledge is relevant for all students, those who later refer patients from other departments as well as for those interested in RO. The student participants agreed that a revision of the concept of QB 11 is necessary for efficient education in RO. The blurring of boundaries between the subjects of QB 11 caused discussion in the focus group. While some wished for closer cooperation, others rather demanded a clear demarcation. Usually, students do not acquire the necessary knowledge for the diagnosis and treatment of tumors in early stages of their studies. However, this is essential for effective teaching in RO which is why students can benefit from integration of RO into a longitudinal–interdisciplinary oncology subject. So far, such a subject has been established at only 41.7% of the faculties in Germany [5, 8, 21, 22]. RT basics should be taught at an early stage in subjects such as biology, physics, and anatomy [5]. Oertel et al. demonstrated the feasibility of introducing RO in the preclinical part of medical education within an “Anatomy and Imaging” course [23]. Zaorsky et al. have already shown that a separate mandatory rotation in RO could reduce knowledge gaps of physicians regarding the indication for radiotherapy or its toxicity [24].

DEGRO experts support, and partly already offer, e.g., mentoring and supervision of scientific work (doctoral programs) or opportunities to join a specific section during Final Medical Year (Praktisches Jahr) [5]. Innovative and attractive formats oriented towards the needs of students are necessary to promote their interests and strengthen them in their development. Students are mainly guided by personal interests, job opportunities, role models, structured training, and salary when choosing their future profession [25–27]. Radiation oncologists accounted for less than 1% of 385,149 physicians in 2017; close to 300 physicians with the specialty designation are in retirement [28]. RO society needs to retain medical students by providing sufficient information and guidance. This should be used as an opportunity and goal to attract future physicians to RO at an early stage in their studies [26, 27, 29].

Curricular renewal is not only about factual knowledge in the education of future physicians, but also about competencies that should be acquired during studies [30]. The Canadian Medical Education Directives for Specialists (CanMEDS) published the concept of CanMEDS Roles in 1996 [31]. Each of these CanMEDS Roles is based on key competencies that must be applied in that role [31]. This concept, originally developed for specialist training, is found in the education of young doctors worldwide [32]. Winseman et al. showed personal and professional growth to be the most important factors affecting empathy in medical education [2]. The wish to be educated and to represent the future role of a communicator was highly present in the focus group. It remains to be discussed and further examined whether students in general are overburdened in this role or whether they see this competence undervalued specifically in RO.

**Fig. 1:**
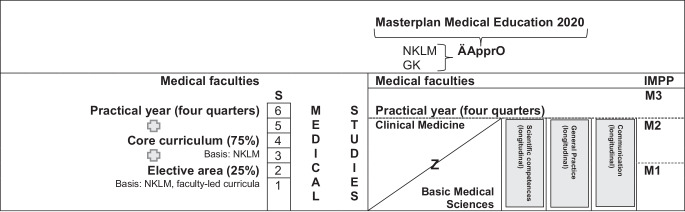
Curricular organization of medical studies taking into account the Z model and institutional responsibilities. *GK* Subject Catalogue, *NKLM* National Competence-Based Learning Objectives Catalogue for Medicine 2.0, *M1 to 3* State examination 1 to 3, *S* Study year

In agreement with the results of our focus group survey, the MFT and GMA call for asynchronous teaching–learning formats that enable self-directed learning [33]. Nonetheless, the effectiveness and use of synchronous interactive methods that benefit from direct exchange and diversity continues to be referenced. The Council of Science and Humanities (Wissenschaftsrat) generally demands that students be given sufficient space to design their elective area individually [34]. This part will make up 25% of future curricula and can be designed by students depending on their interests (Fig. 1). The draft of ÄApprO aims to teach the competent use of digital technologies. MFT and GMA support the use of digital formats as a supplement to established face-to-face teaching [33]. This was also confirmed by the recently published study results of Vorwerk et al. in the field of RO [35]. Oertel et al. also demonstrated the successful digital transfer of a core curriculum in RO fastened by SARS-CoV‑2 pandemic [36]. A study at the Technical University of Munich showed how one-dimensional formats in teaching can be supplemented by the flipped classroom principle to achieve an increase in the internalization of content [37]. In contrast to classic lectures, students come to the classroom already prepared and learn how to acquire knowledge themselves [38]. They train to work as a team to solve complex problems [39]. A study in the Republic of Korea examined how this model could be implemented in RO teaching [40]. In a pilot project, students received instructional videos in preparation for a thematically linked discussion on the following day. In a final questionnaire survey, the students showed a high level of motivation and satisfaction. However, aforementioned model shows weaknesses because such methods rely on the cooperation and motivation of each student [37, 39]. Modern teaching instruments are partly already integrated as in the ZOOM software or available online as a freeware tool (e.g., Mentimeter) and are easy to use [41, 42]. Possibility of digital teaching formats, anchoring of NKLM as a core curriculum and linking of theoretical and clinical content within the framework of a Z-curriculum were evaluated positively by the students. The Z‑curriculum describes an interweaving of basic, clinical–theoretical and clinical–practical contents over the entire duration of the study program. The respective parts shift from the basics to clinical activity (Fig. 1). Overall, different teaching formats should be combined for varied and efficient teaching. At this point, the German Medical Association (Bundesärztekammer*)* and the German Association of University Medicine (Deutsche Hochschulmedizin) have expressed their concerns over the increase in number of compulsory hours required for implementation after final development and revision of Masterplan Medical Education 2020: It will definitely lead to an overload of medical studies [43–45]. Rabatta et al. stated that this will not only lead to a higher burden on students, but also challenge personnel/staff and finances. In addition, a fully developed financing plan for the restructuring is still missing [33].

Of note, our prospective study has some limitations. The study team exclusively used a qualitative, descriptive study design and did not intend to investigate quantitative data. Nevertheless, in-depth insights into the students’ experiences and the named needs and strategies point towards important aspects. However, the study was concerned with improving teaching in RO, so it can be assumed that participants are particularly interested in RO or at least more interested than average in improving teaching on the federal level. In the context of compulsory university courses, less motivated and interested students are to be expected. A needs analysis in a larger group of students that is not specifically interested in RO could therefore be necessary in the future. Furthermore, participants showed a wide range regarding medical knowledge due to a high heterogeneity in semester number. Some participants have not yet attended any or only some of the courses in RO; specific questions regarding the evaluation of some courses could therefore not be answered by them. Nevertheless, their insights affixed valuable additions. Finally, all participants were students at the Medical Faculty of the University of Cologne, so that only limited conclusions can be drawn about teaching on a federal level.

This study was intended to be exploratory and to yield suggestions for further research, rather than for instructional practice. Nevertheless, this research supports the improvement of medical teaching in RO. Further prospective quantitative and qualitative studies that consider each location of (university) medicine are needed to evaluate students’ needs in RO teaching in Germany and to optimize the upcoming curriculum of new ÄApprO, underlined, and focused by *Vision 2030 for radiotherapy & radiation oncology in Germany* [46, 47].

## Conclusion

The present work is intended to support the integration of RO into the compulsory teaching of medical students in Germany. Longitudinal training of students on the possibilities and importance of RO as one of the leading players of tumor therapy should be the goal of curriculum development. The results of the needs analysis for the subject of RT are consistent with ÄApprO, NKLM, and DEGRO experts. Moreover, they complement them and should be considered in curriculum development of Masterplan Medical Education 2020. The results contribute to high-quality and target-group-oriented medical training in the subject of RO, increased visibility, and thus early attachment of future physicians to RO in Germany.

## Abbreviations

ÄApprO: Licensing Regulations for Physicians; Ärztliche Approbationsordnung
CanMEDS: Canadian Medical Education Directives for Specialists
DEGRO: German Society for Radiation Oncology; Deutsche Gesellschaft für Radioonkologie
GK: Subject Catalogue; Gegenstandskatalog
GMA: Society for Medical Education; Gesellschaft für Medizinische Ausbildung
IMPP: Institute for Medical and Pharmaceutical Examination Questions; Institut für medizinische und pharmazeutische Prüfungsfragen
MFT: Medical Faculty Association of the Federal Republic of Germany; Medizinischer Fakultätentag der Bundesrepublik Deutschland
NKLM: National Competence-Based Learning Objectives Catalogue for Medicine 2.0; Nationaler Kompetenzbasierter Lernzielkatalog Medizin 2.0
PBL: Problem-based learning
QB 11: Cross-sectional subject 11. Imaging procedures, radiation therapy, radiation protection; Querschnittsbereich 11: Bildgebende Verfahren, Strahlenbehandlung, Strahlenschutz
RO: Radiation oncology
RT: Radiation therapy
